# Unraveling the role of the gut microbiome in pregnancy disorders: insights and implications

**DOI:** 10.3389/fcimb.2025.1521754

**Published:** 2025-03-07

**Authors:** Yupei Xie, Qian Chen, Dan Shan, Xiongfei Pan, Yayi Hu

**Affiliations:** ^1^ Department of Obstetrics and Gynecology, West China Second University Hospital, Sichuan University, Chengdu, China; ^2^ Key Laboratory of Birth Defects and Related Diseases of Women and Children, Sichuan University, Ministry of Education, Chengdu, China; ^3^ Department of Obstetrics and Gynecology, Qingbaijiang Maternal and Child Health Hospital, Chengdu, China; ^4^ West China Second University Hospital, Sichuan University, Shuangliu Institute of Women’s and Children’s Health, Shuangliu Maternal and Child Health Hospital, Chengdu, China

**Keywords:** gut microbiota, preeclampsia, insulin resistance, probiotics, postpartum depression, gestational diabetes mellitus

## Abstract

The gut microbiota is the collective term for the microorganisms that reside in the human gut. In recent years, advances in sequencing technology and bioinformatics gradually revealed the role of gut microbiota in human health. Dramatic changes in the gut microbiota occur during pregnancy due to hormonal and dietary changes, and these changes have been associated with certain gestational diseases such as preeclampsia (PE) and gestational diabetes mellitus (GDM). Modulation of gut microbiota has also been proposed as a potential treatment for these gestational diseases. The present article aims to review current reports on the association between gut microbiota and gestational diseases, explore possible mechanisms, and discuss the potential of probiotics in gestational diseases. Uncovering the link between gut microbiota and gestational diseases could lead to a new therapeutic approach.

## 1 Introduction

Pregnancy is a normal physiological phenomenon, during which the major organs of the body undergo a series of adaptive changes to meet the needs of pregnancy (Ouzounian and U, 2012). Successful pregnancy is influenced by many factors such as environmental pollution, poor lifestyle habits, and others (Thiele et al., 2019). At the same time, pregnancy is considered an allograft and immune tolerance between maternal tissue, placenta and fetus is essential for a successful pregnancy (Goldstein et al., 2020). Extensive clinical and basic research has made great strides in understanding the various factors that contribute to a successful pregnancy. However, changes in the gut microbiota during pregnancy have been overlooked by clinicians and researchers for many years. In fact, the gut microbiota also plays an important role in successful pregnancy, and gut dysbiosis has been shown to be associated with many pregnancy complications (Ziętek et al., 2021).

**Figure 1:**
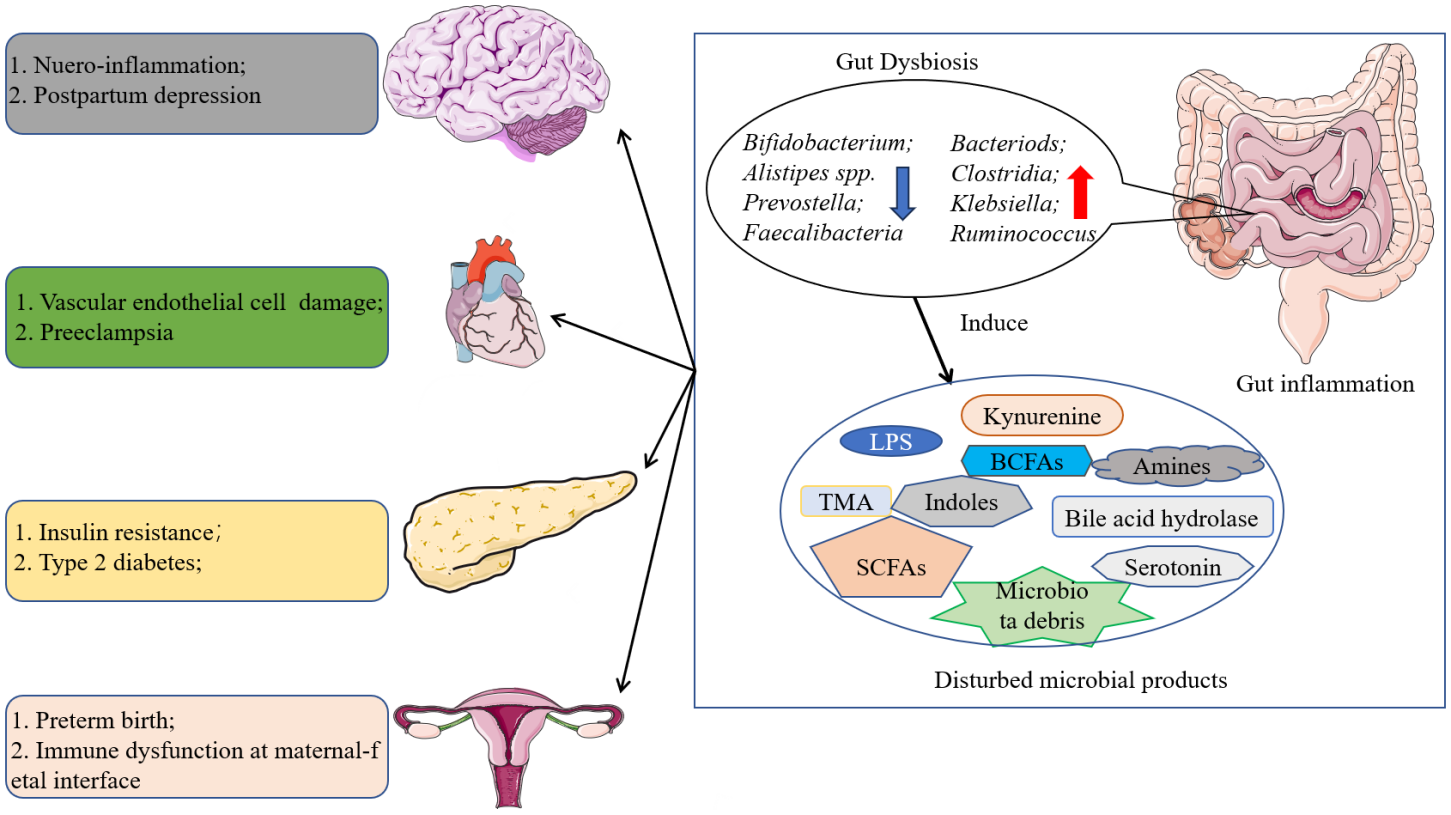
Role of gut microbiota in organs dysfunction. The disturbed gut microbiota may induce lot kind of diseases by modulating the metabolism of fatty acids and amino acids and so on, and further leading to auto-immune disease and metabolism related diseases.

Despite the current sophisticated healthcare measures during pregnancy, the incidence of diseases in pregnancy (especially metabolic diseases) continues to be high as living standards improve. For instance, the global prevalence of gestational diabetes mellitus (GDM), a classic metabolic disorder, is approximately 14% and has an impact on pregnancy outcomes (Wang et al., 2022). Gut microbiota is the general term for microbes that colonize the human gut. Benefiting from the Human Microbiome Project, the composition of gut microbiota and its role in human health has been gradually reported (Consortium., I H i R N, 2019). Indeed, the gut microbiota of healthy individuals always have higher diversity and predominant phyla are *Firmicutes*, *Actinobacteria*, *Bacteroidetes*, and *Proteobacteria*. Human body may suffer a series of immune- or metabolism-related diseases when gut dysbiosis occurs (G, 2020). Mechanistic research revealed that an increase in the relative abundance of opportunistic pathogenic bacteria and a decrease in the relative abundance of probiotic bacteria are thought to induce or promote disease progression by promoting inflammation, increasing toxic metabolites, increasing intestinal epithelial permeability, and altering crosstalk between the intestine and individual organs such as the brain and liver. Moreover, the gut microbiota was supposed to be associated with gestational diseases such as preeclampsia (Chen et al., 2020), gestational diabetes mellitus (Medici Dualib et al., 2021), and postpartum depression (Hou et al., 2020) in **Figure 1**. For example, the over proliferation of *Proteobacteria* and the progressive decrease of *Firmicutes* in the intestinal flora are thought to be associated with the development of GDM, and the relative abundance of *Neiss./Lepto.* and *Prevo./Aeroc.* has been shown to be positively correlated with OGTT test values (Wang et al., 2018). Likewise, higher abundance of *Bacteroidetes*, *Proteobacteria*, and *Actinobacteria* was observed in the intestinal flora of preeclampsia patients, and these alterations in intestinal bacteria are thought to be associated with higher serum concentrations of toxic bacterial products such as LPS and TMAO (Wang et al., 2019). These studies imply that gut microbiota is inextricably linked to gestational diseases and elucidating the involved pathogenic mechanisms is essential for their prevention and treatment. Given the important role that gut microbiota plays in health, this review aims to summarize the currently published literature on the relationship between gut microbiota and gestational diseases, and discuss the potential mechanism of microbe-host interaction during pregnancy. It is expected to provide a new perspective for future research on gut microbiota and pregnancy diseases as well as the clinical application of gut microbiota intervention.

## 2 Gut microbiota shifts in pregnant women

In general, the gut microbiota of healthy adults can be mainly divided into four categories, namely *Bacteroidetes*, *Firmicutes, Actinobacteria* and *Proteobacteria*, which account for 23%, 64%, 3% and 8%, respectively (Adak and K, 2019). The structure of normal intestinal flora can be affected by changes in hormone levels. A study comparing the differences in gut microbial structure between uncastrated male mice, castrated male mice and female mice showed that the structure of gut flora was most similar in castrated male mice and female mice (Yurkovetskiy et al., 2013). Conversely, the intestinal flora also affects the level of hormones in the body, and this phenomenon is related to the deconjugation of food-derived hormones by β-glucuronidase synthesized by the intestinal flora (He et al., 2021). Nevertheless, the impact of fluctuations in estrogen and progesterone levels on the gut microbiota of women during the natural menstrual cycle remains somewhat controversial. Mihajlovic et al. compared gut microbiota diversity during the follicular phase (when serum sex hormone levels are relatively low) and the luteal phase (when serum sex hormone levels are relatively high) in 9 women aged 21-29. The results indicated that fluctuations in serum sex hormone levels throughout the menstrual cycle were insufficient to influence β-diversity of gut microbiota (Mihajlovic et al., 2021). Another study involving 20 Belgian women of reproductive age continuously sampled their gut microbiota over a period of six weeks and conducted microbiome analysis. The results showed that the majority of gut microbes exhibited significant temporal variation, with some bacterial genera experiencing over a 100-fold change in abundance during the study period. Furthermore, the diversity and evenness indices of the participants’ gut microbiota fluctuated considerably. However, researchers believe that these changes in the gut microbiota are unrelated to the fluctuations in sex hormone levels during the menstrual cycle (Vandeputte et al., 2021). Although the aforementioned studies all indicate that gut microbiota diversity does not change with hormonal fluctuations during the menstrual cycle, both studies note an increase in the relative abundance of *Akkermansia* during the luteal phase when hormone levels rise.

Similarly, changes in the gut microbiota during pregnancy have been reported (Nuriel-Ohayon et al., 2019; Yoon and K, 2021), with alterations of the intestinal flora occurring primarily during the second and third trimesters in response to increasing levels of relevant hormones (Sun et al; Smid et al., 2018). Studies have demonstrated that during early pregnancy, the gut microbiota remains predominantly dominated by *Firmicutes*, exhibiting consistent stability compared to the pre-pregnancy period (Koren et al., 2012). As pregnancy progresses, gut microbiota changes are primarily characterized by reduced α-diversity (within hosts), and increased dissimilarity between hosts (β-diversity) (Nuriel-Ohayon et al., 2016). The relative abundances of butyrate-producing *Bifidobacteria* and lactic acid-producing microorganisms increase (Ruebel et al., 2021), as do the relative abundance of *Akkermansia* (Qin et al., 2021), similar to changes during the menstrual cycle, potentially associated with elevated sex hormones during pregnancy. These changes in gut microbiota are thought to drive the host to store sufficient energy, maintain insulin resistance, and ensure relatively high blood glucose levels to meet the developmental needs of the fetus, placing the host in a state of metabolic conditions akin to diabetes (Koren et al., 2012; Gomez-Arango et al., 2016a; Ziętek et al., 2021). Researchers provided experimental evidence for this theory by transplanting the gut microbiota of late-term pregnant women into germ-free mice; glucose metabolism disorders were observed in the mice that received the microbiota transplant (Koren et al., 2012). Moreover, as pregnancy progresses, the abundance of *Actinobacteria* and *Proteobacteria* increases, and microbial communities associated with short-chain fatty acid and butyrate production decrease to some extent (Koren et al., 2012). Adaptive adjustments in the gut microbiota occur during normal pregnancy are thought to serve several important functions, including promoting fetal growth and development, reducing infection risk in the host, and optimizing the host’s nutritional metabolism (Sinha et al., 2023).

During pregnancy, various factors other than hormones can also influence the gut microbiota. Diet is a key factor that can impact the gut microbiota, whether pregnant or not (Barrett et al., 2018). A diet high in fat and salt, as well as the intake of trace elements and dietary fiber, can all influence gut microbiota (Gioia et al., 2020). Guo et al. studied the effects of a high-salt diet on gut microbiota and reported that a high-salt diet increased the relative abundance of *Proteobacteria* and *Bacteroides* (Guo et al., 2021). The effects of alcohol consumption on maternal gut microbiota during pregnancy were also studied, with alcohol consumption positively correlated with *Phascolarctobacterium* and *Blautia* and negatively correlated with *Faecalibacterium* (Wang et al., 2021). In addition, the ingestion of exogenous bacterial preparations during pregnancy can alter gut microbiota structure. A study of mice administered *Akkermansia muciniphila* during pregnancy showed that supplementation can alter diversity and composition of the gut microbiota and strengthen the gut barrier (Qi et al., 2022). Furthermore, immune changes (F, 2020), antibiotic intervention (Zhou et al., 2020; Benner et al., 2021), sleep factors (Yao et al., 2022), and psychological stress (Hechler et al., 2019) can all influence gut microbiota composition in humans and animals. It is clear that the gut microbiota of pregnant women can change during pregnancy.

## 3 Gut microbiota and gestational diabetes mellitus

Women experience major metabolic changes from preconception through pregnancy, primarily manifested as enhanced anabolic metabolism, elevated serum free fatty acid and steroid hormone levels, and decreasing amino acid levels. Additionally, fasting blood glucose decreases in the early stages of pregnancy, while a degree of insulin resistance may occur in the second and third trimesters (Hadden and M, 2009; Liang et al., 2020). All these pregnancy-related metabolic changes typically return to pre-pregnancy levels shortly after childbirth. Gestational diabetes mellitus (GDM) refers to a disorder of maternal blood glucose metabolism first diagnosed during pregnancy (Association, A D, 2019). In terms of pathophysiology, the main aberrations in GDM are chronic inflammation, insulin resistance, and pancreatic islet β cell dysfunction (Plows et al., 2018). It has numerous effects on the mother and fetus, including abnormal fetal development, polyhydramnios, infections, and fetal macrosomia and dystocia (Szmuilowicz et al., 2019). The impact of gestational diabetes mellitus on mothers does not appear to be limited to pregnancy. Studies have shown that patients with gestational diabetes mellitus have a 10-fold higher risk of developing type 2 diabetes later in life (Vounzoulaki et al., 2020), and it can also program the metabolism of offspring and severely affect their health (Chen et al., 2018). So far, insulin is still the most effective therapy, but it can sometimes cause side effects. Therefore, finding a new target to prevent it is of great importance.

In recent years, there has been an increase in research on the gut microbiota, revealing differences in the gut microbiota of patients with and without gestational diabetes mellitus (GDM) and their putative roles (shown in **Table 1**). Wei et al. compared the gut microbiota of GDM patients (n=15) with that of healthy controls (n=18), reporting differences in β diversity of the gut microbiota and a higher abundance of *Ruminococcus bromii*, *Clostridium colinum*, and *Streptococcus infantis* in GDM patients. Among these altered bacteria, *Streptococcus infantis* was positively associated with a higher risk of GDM (Wei et al., 2022). Another study also reported differences in β diversity while several altered bacteria including *Bacteroides dorei* and *Bacteroides* spp. 3_1_33FAA, were negatively associated with glucose tolerance, and *Alistipes putredinis* positively related to insulin sensitivity (Wu et al., 2020). Although similar studies have reported differences in the gut microbiota of GDM patients and healthy pregnant women, the specific altered bacteria have not been consistent, possibly due to different study methods and subjects (Medici Dualib et al., 2021). To better elucidate the causal relationship between gut microbiota and GDM, Sun et al. compared the gut microbiota of GDM patients (n=120) before disease onset with that of healthy pregnant women (n=120) of the same gestational age, reporting differences existed before disease onset. Compared to the control group, during pregnancy, the relative abundance of *Ruminococcus bromii* was consistently lower in patients with GDM. Moreover, *Desulfovibrio* and *Bacteroides ovatus* also remained lower at T1 and T2. The study also indicates that gut fiber fermentation metabolism is associated with blood glucose control in patients with gestational diabetes (Sun et al). Furthermore, transplantation of the gut microbiota of GDM patients to germ-free mice developed abnormal blood glucose metabolism, supporting a causal relationship (Liu et al., 2020). This result provides strong evidence for a causal relationship between gut microbiota and GDM, and demonstrate that the gut microbiota of different types exerts distinct impacts on the occurrence of GDM. Moreover, one study reported changes in the gut microbiota of GDM patients following metformin therapy, suggesting that this change may represent a therapeutic mechanism of metformin (Molina-Vega et al., 2022). Diet modification (Ponzo et al., 2019), exercise increases (Mahizir et al., 2020), probiotics consumption (Zheng et al., 2021), and trace elements supplementation (Zhang et al., 2021) have all been reported to improve glucose metabolism in GDM patients by modulating or restoring gut microbiota. Additionally, some researchers utilized mendelian randomization, a causal inference analytical technique, to investigate potential causal associations between differences in gut microbiota and GDM at the genetic level. Study results indicate that an increased relative abundance of *Collinsella*, *Coprobacter*, *Olsenella*, *Lachnoclostridium*, *Prevotella 9*, and *Ruminococcus 2* may be associated with an increased risk of GDM. Conversely, the abundance of *Oscillibacter* and *Methanobrevibacter* is negatively correlated with the risk of gestational diabetes mellitus. In addition, this study reveals that gut microbiome metabolites significantly associated with an increased risk of GDM include serine, indole, acetate, adrenate, and phenylacetate; while metabolites such as pyruvate, pipecolate, glycodeoxycholate, and carnitine demonstrate potential protective effects against GDM (Wu et al., 2023b). Although the reported gut microbiota-GDM relationship offer a new perspective, the primary task remains clarifying potential mechanisms by which gut microbiota contributes to insulin resistance, thereby better exploiting the gut microbiota as a strategy for treating or preventing GDM.

**Table 1:** Clinical studies on gut microbiota and gestational diabetes mellitus and preeclampsia.

Diseases	Study design	N (cases: controls)	Sequence strategy	Sampling gestational age (weeks)	Microbiota enriched	Microbiota decreased	Refs
GDM	prospective	15: 18	16S	24-28	*Clostridiales*, *Clostridia*, *Firmicutes*	*Bacteroidales*	(Wei et al., 2022)
	prospective	98: 98	16S(V4)	10-14	*Eisenbergiella*, *Tyzzerella* 4, *Lachnospiraceae* NK4A136	*Parabacteroides*, *Megasphaera*, *Eubacterium eligens group*	(Ma et al., 2020)
	case control	11: 11	16S (V3-V4)	27-33	*Actinobacteria*	/	(Liu et al., 2019)
	case control	21: 32	16S (V3-V4)	24-28	*Corynebacteriales* (order), *Nocardiaceae* (family), *Desulfovibrionaceae* (family), *Rhodococcus* (genus), *Bacteroidetes* (phylum)	/	(Su et al., 2021)
	case control	59: 48	16S (V3-V4)	24-28	family *Lachnospiraceae*	families *Enterobacteriaceae*, *Ruminococcaceae*	(Wang et al., 2020)
	case control	30: 28	16S rRNA microarray	24-28	*Species: Aureimonas altamirensis*,*Kosakonia cowanii*	Phylum: *Prevotella*, *Romboutsia*	(Chen et al., 2021)
	case control	201: 201	16S (V3-V4)	6-15 & 24-28	*Enterobacteriaceae*, *Ruminococcaceae* spp., *Veillonellaceae*	*Rothia*, *Actinomyces*, *Bifidobacterium*, *Adlercreutzia*, *Coriobacteriaceae*, *Lachnospiraceae* spp.	(Hu et al., 2021)
	prospective	case 41	16S	24-28 & 38	*Firmicutes*	*Bacteroidetes, Actinobacteria*	(Ferrocino et al., 2018)
	case control	49: 42	16S	24-28 & 34-38	unidentified_*Lachnospiraceae*, *Blautia*, *Parabacteroides*	*Bifidobacterium*	(Liu et al., 2023)
	case control	43: 81	whole-metagenome shotgun	21-29	*Parabacteroides distasonis, Klebsiella variicola, etc.*	*Methanobrevibacter smithii, Alistipes* spp.*, Bifidobacterium* spp.*, Eubacterium* spp.	(Kuang et al., 2017)
	case control	35: 25	16S (V4)	24-28	*Bacteroides, Lachnoclostridium*	*Ruminococcaceae*_UCG-002, *Ruminococcaceae*_UCG-005, *Clostridium_sensu_stricto*_1, *Streptococcus*	(Liang et al., 2022)
	case control	30: 31	16S	/	*Gammaproteobacteria*, *Hemophilus*	/	(Xu et al., 2020)
	case control	50: 157	16S (V1-V2)	27–33	*Faecalibacterium, Anaerotruncus*	*Clostridium* (sensu stricto), *Veillonella*	(Crusell et al., 2018)
	prospective	44: 350	16S (V4)	11-13^+6^	/	*Prevotella*	(Pinto et al., 2023)
	prospective	31: 103	16S (V3-V4)	8-12 & 24-28	/	*Coprococcus,Streptococcus*	(Zheng et al., 2020)
	case control	53: 16	16S	24-28 & 36-40	*Collinsella*, *Blautia*, *Ruminococcus*, *gnavus Ruminococcus*, *torques Ruminococcus*, *Eubacterium hallii*	/	(Gupta et al., 2024)
	case control	56: 59	16S	/	*Bacteroides*	/	(Dualib et al., 2022)
	case control	30: 11	16S (V3)	/	*Blautia*	*Sphingomonas (Proteobacteria)*	(Benítez-Guerrero et al., 2022)
	prospective	26: 12	16S (V3-V4)	T1 & T3	*Acidaminococcus*, *Clostridium*, *Megasphaera*, *Allisonella*	*Blautia*	(Abdullah et al., 2022)
	case control	12: 10	16S	/	*Streptococcus*	*Roseburia*	(Shen et al., 2024)
	case control	20: 20	16S (V3-V4)	median: 34	*Phascolarctobacterium*, *Alistipes*, *Parabacteroides*, *Eubacterium coprostanoligenes*_group, *Oscillibacter*, *Paraprevotella*, *Ruminococcaceae* NK4A214_group	*Blautia*	(Dong et al., 2020)
	case control	50: 54	Metagenomic	/	/	*Faecalibacterium, Prevotella, Streptococcus*, sp*ecies Bacteroides coprophilus, Eubacterium siraeum, Faecalibacterium prausnitzii, Prevotella copri, Prevotella stercorea*,	(Ye et al., 2023)
	case control	110: 220	16S	22-24	*Bacteroidetes*	*Firmicutes, Actinobacteria*	(Chen et al., 2021)
	case control	51: 22	16S	T1 & T3	genera: *Enterococcus, Erysipelotrichaceae UCG*-003	family *Prevotellaceae*, order *Fusobacteriales*, genus *Sutterella*.	(Vavreckova et al., 2022)
	case control	27: 30	16S	/	phyla *Firmicutes, Bacteroidetes, Proteobacteria, Lentisphaerae*	/	(Wu et al., 2022)
	case control	20: 16	16S	T1, T2 & T3	genus: *Romboutsia*	*Prevotella 9*	(Tanaka et al., 2022)
Preeclampsia	case control	12: 8	16S (V4)	/	genus *Blautia, Ruminococcus*	phylum *Actinobacteria*, family *Bifidobacteriaceae*, genus *Bifidobacterium*	(Miao et al., 2021)
	case control	40: 37	metagenomic	T3	*Blautia*, *Pauljensenia*, *Ruminococcus*, *Collinsella*	*Bacteroides*, *Phocaeicola*	(Lv et al., 2022)
	case control	78: 72	16S	T3	*Blautia, Ruminococcus*2*, Bilophila, Fusobacterium*	*Faecalibacterium, Gemmiger, Akkermansia, Dialister, Methanobrevibacter*	(Lv et al., 2019)
	case control	67: 85	16S	/	*Clostridium, Dialister, Veillonella, Fusobacterium*	*Faecalibacterium, Akkermansia*	(Chen et al., 2020)
	case control	25: 29	16S	T3	*Bacteroidetes*	phylum: *Verrucomicrobia, Syntergistota; genus: Akkermansia*	(Meijer et al., 2023)
	case control	48: 48	16S (V4)	/	*Bacteroides_fragilis*	genera *Firmicutes,Clostridia, Clostridiales, Ruminococcaceae, Rikenellaceae,Faecalibacterium, Alistipes, Bacteroides_stercoris*	(Wang et al., 2019)
	case control	25: 25	16S (V4)	T2 & T3	*Bacteroidetes, Proteobacteria, Enterobacteriaceae*	*Firmicutes*	(Wang et al., 2020)
	case control	6: 9	16S	27-40	*Rothia, Actinomyces, Enterococcus*	*Coprococcus*	(Wu et al., 2024)
	case control	17: 30	16S	/	*Polycyclovorans*, *Pelomonas*	*Thermomonas, Xanthomonas, Methanobrevibacter*	(Wu et al., 2023a)
	case control	26: 24	16S	T3	*Clostridiu perfringens, Bulleidia moorei*	*Coprococcus catus*	(Liu et al., 2017)
	case control	27: 36	16S	/	phylum: *Proteobacteria*; genus: *Enterobacter, Escherichia Shigella*	phylum: *Firmicutes*; genus:*Blautia*, *Eubacterium rectale*, *Eubacterium hallii*, *Streptococcus*, *Bifidobacterium*, *Collinsella*, *Alistipes*, Subdoligranulum	(Chang et al., 2020)
	case control	41: 45	16S	/	*Proteobacteria*	*Bacteroidetes*	(Zhao et al., 2023)

**Figure 2:**
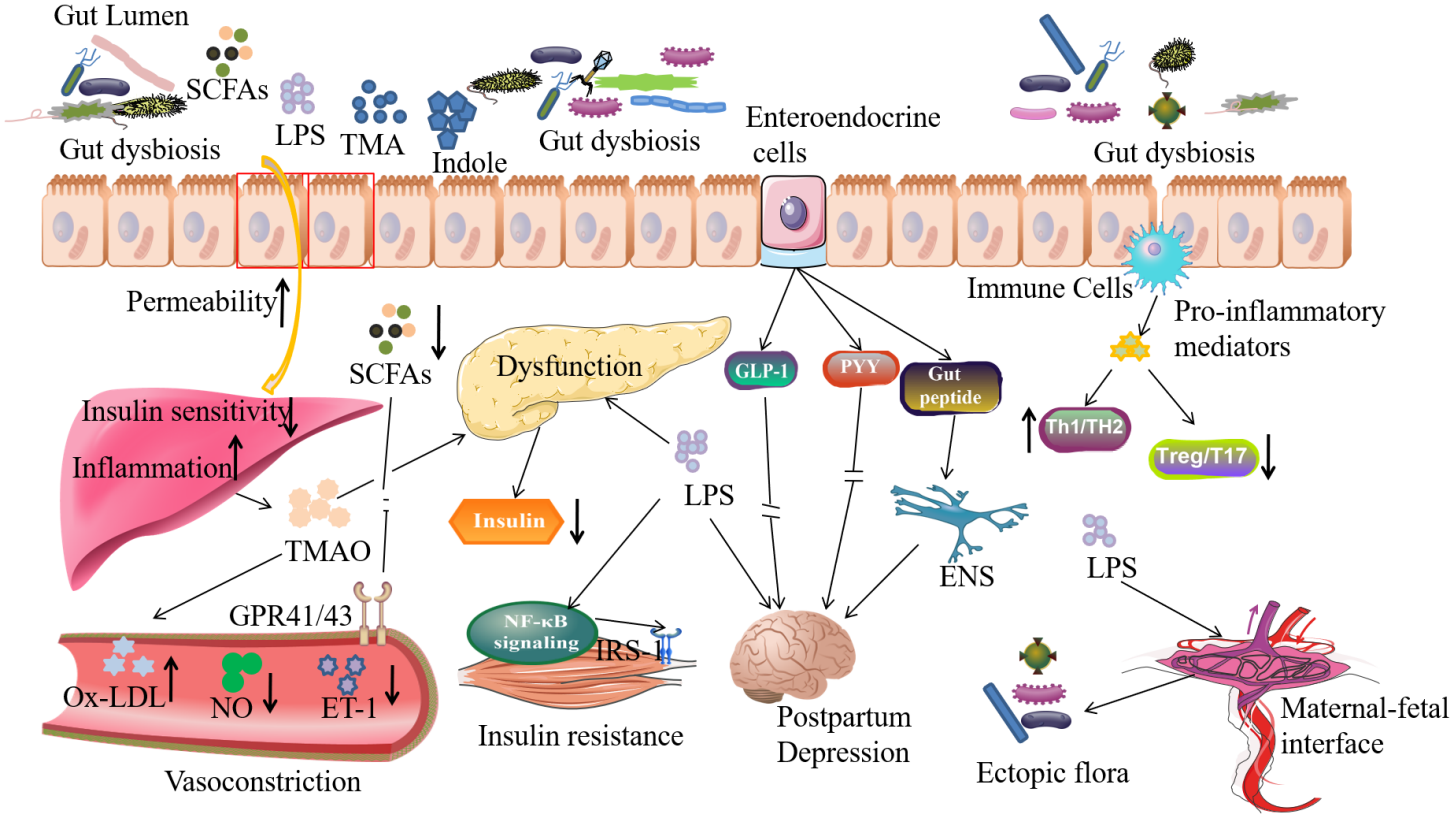
Mechanisms of gut dysbiosis related organ dysfunction. When gut microbiota was disturbed, their structure and biological function may change and produce more detrimental metabolites, such as TMA, indole, etc. which may lead to dysfunction of liver, pancreas, brain, blood vessel and intestine barrier. And then lead to PE, GDM, PPD and preterm labor in pregnancy. SCFAs, short chain fatty acids; LPS, lipopolysaccharide; TMA, trimethylamine; TMAO, trimethylamine N-oxide; GLP-1, glucagon-like peptide 1; PYY, peptide YY; ENS, enteric nervous system; IRS-1, Insulin receptor substrates-1; Ox-LDL, oxidized low density lipoprotein; ET-1, endothelin-1; NO, nitric oxide; GPR41/43, G protein coupled receptors 41/43.

Mechanistically, GDM is characterized by relative insulin insensitivity, which explains the tendency for individuals to develop type 2 diabetes later in life (Vounzoulaki et al., 2020), and may be at the core of the relationship between gut microbiota and GDM. Insulin receptor substrate-1 (IRS-1), a key link in the insulin signaling pathway (Yaribeygi et al., 2019), has been shown to be phosphorylated and inactivated when the NF-κB pathway is activated, resulting in attenuated insulin signaling (Zhang et al., 2018). This is the mechanism by which inflammation impairs tissue insulin sensitivity. Additionally, lipopolysaccharide (LPS), the major component of the cell wall of Gram-negative bacteria, also known as endotoxin, has been shown to activate the NF-κB pathway in type 2 diabetes (Andreasen et al., 2011). In the absence of infection, the gut is considered the primary source of circulating serum LPS. Dysbiosis of the gut microbiota in pregnancy-related disorders can lead to intestinal barrier damage, ultimately resulting in significantly increased serum LPS levels (Huang et al., 2021b). Therefore, it is plausible that excessive activation of the LPS/NF-κB pathway may be one mechanism by which gut dysbiosis during pregnancy contributes to GDM. Moreover, it has been gradually reported that the fermentation products of the gut microbiota play some role in GDM (see in **Table 2**) (Gao et al., 2020a). Short-chain fatty acids (SCFAs), the most studied fermentation products of carbohydrates derived from the gut microbiota, are mainly composed of acetate, butyrate and propionate, and play a vital role in metabolism (Morrison and P, 2016). Recent studies have reported that SCFAs promote the secretion of glucagon-like peptide 1 (GLP-1) and peptide YY (PYY) by activating intestinal receptors GPR41/43, thereby increasing tissue sensitivity to insulin (Kimura et al., 2014). SCFAs have also been shown to play a role in maintaining intestinal epithelial barrier function, and a deficiency of SCFAs can lead to an excessive influx of LPS that eventually causes inflammation, or directly result in decreased secretion of anti-inflammatory mediators, disrupting glucose metabolism (Martin-Gallausiaux et al., 2021). SCFAs can even alter energy expenditure by affecting the activity of uncoupling proteins (He et al., 2020). In addition to SCFAs, amino acid metabolism is also involved in the interaction between gut dysbiosis and insulin resistance (Zhang et al., 2019). A normal gut microbiota can convert aromatic amino acids to indoles, which has been reported to play a protective role in maintaining the intestinal barrier and promoting GLP-1 secretion, thereby improving tissue insulin sensitivity (Canfora et al., 2019). Trimethylamine n-oxide (TMAO), the product of choline fermentation by gut microbiota, has also been shown to contribute to the regulation of metabolic function (Day-Walsh et al., 2021). It can trigger endoplasmic reticulum stress and lead to tissue remodeling disorders (Govindarajulu et al., 2020). Further, Yang et al. performed cell assemblies and reported that TMAO promotes apoptosis of pancreatic acinar cells by activating the expression of endoplasmic reticulum stress signaling pathway (Yang and Z, 2021). However, Krueger et al. concluded to the opposite, that TMAO has some protective effect on pancreatic β cell damage and islet dysfunction caused by type 2 diabetes-like glucolipotoxicity (Krueger Es et al., 2021). Moreover, TMAO may also induce inflammation via the NF-κB pathway, which has been reported to block insulin signaling (Yang G et al., 2019). Combined with the above studies, despite inconsistent results, we still conclude that the gut microbiota plays a pivotal role in GDM (**Figure 2**).

**Table 2:** Reported metabolites of gut microbiota associated with gestational diabetes mellitus and preeclampsia.

Diseases	Subjects	Metabolites	Potential mechanism	Ref.
GDM	Homo	Increased: Isobutyric acid, Isovaleric acid, Valeric acid, Caproic acid, GUDCA, THDCA + TUDCA, LCA-3S	/	(Gao et al., 2020a)
	Homo	Increased: Iso-butyrate; Decreased: Butyrate	/	(Han et al., 2024)
	Homo	Decreased: Acetate, propionate, butyrate	/	(Zheng et al., 2022)
	Homo	Increased: butyrate, mevalonate	Impacting the metabolism of pregnancy hormones	(Lyu et al., 2023)
	Homo	Increased: hydrocinnamic acid, 3-(4-hydroxyphenyl) propionic acid, 4-hydroxyphenylacetic acid, isoleucine	promoting insulin resistance	(Susarla et al., 2024)
	Homo	Increased: phenylalanine	/	(Lin et al., 2024)
Preeclampsia	Homo	Decreased: acetate	impairing fetal thymic and regulatory T cell	(Hu et al., 2019)
	Homo	Decreased: Butyrate	/	(Gomez-Arango et al., 2016b)
	Homo	Increased: lipopolysaccharide (LPS), trimethylamine-N-oxide (TMAO)	/	(Wang et al., 2019)
	Homo & Mice	Increased: Trimethylamine N-oxide (TMAO)	affecting the migration and angiogenesis of vascular endothelial cells	(Wang et al., 2024)
	Homo & Rat	Decreased: propionate, butyrate	promoting autophagy and M2 polarization of macrophages in placental bed	(Jin et al., 2022)
	Homo	Increased: Phenylpropanoate, Agmatine	/	(Liu et al., 2024)
	Homo	Dcreased: betaine; Increased: trimethylamine oxide (TMAO)	/	(Xu et al., 2024)
	Homo & Rat	Decreased: butyric, valeric acids	reducing the blood pressure	(Chang et al., 2020)
	Homo	Increased: acetate, propionate, isobutyrate, valerate; Decreased: caproic acid, butyrate	/	(Li et al., 2022)

## 4 Gut microbiota and preeclampsia

Pre-eclampsia (PE), with a global prevalence of 3-5%, is a disorder of maternal blood pressure regulation during pregnancy, characterized by hypertension and proteinuria, posing serious threats to the life of the mother and fetus. Its pathogenesis remains unclear, and the current prevailing theory suggests that inadequate remodeling of the uterine spiral arterioles, excessive immune activation, vascular endothelial damage, nutritional deficiencies, and genetic factors may all be involved (Ramos et al., 2017). To better manage the disease, pathophysiological changes of preeclampsia are divided into two stages: the first due to dysregulated production of immunoregulatory cytokines and angiogenic factors leading to poor trophoblast invasion; the second characterized by a maternal inflammatory response, systemic inflammatory syndrome (Rana et al., 2019). Early diagnosis and treatment is key, but prevention and treatment based on currently established high-risk factor prediction systems has been ineffective, and it is crucial to identify other more sensitive biomedical markers (Ma’ayeh and C, 2020). Recently, research on gut microbiota-human health interaction has brought certain possibilities for preeclampsia diagnosis and treatment, with differences found in gut microbiota between preeclamptic and normal pregnant women suggesting the potential for the gut microbiota as an early diagnosis biomarker (Lv et al., 2019).

Miao et al. reported decreased relative abundance of *Actinobacteria*, *Bifidobacteriaceae* and *Bifidobacterium* at the phylum, family, and genus levels, respectively, and increased abundance of the genera *Blautia* and *Ruminococcus* in the gut microbiota of preeclampsia patients compared to controls. Correlation analyses confirmed that *Blautia* and *Ruminococcus* were positively correlated with obesity and dyslipidemia, high-risk factors for preeclampsia (Miao et al., 2021). In addition, higher abundance of *Blautia, Ruminococcus, Bilophila*, and *Fusobacterium* and relatively low abundance of *Faecalibacterium, Gemmiger, Akkermansia, Dialister*, and *Methanobrevibacter* were reported in the prenatal gut microbiota of patients with early-onset preeclampsia (occurring between 20 and 34 weeks of gestation) (Lv et al., 2019). Studies also reported other bacteria changes in preeclampsia (see in **Table 1**) (Liu et al., 2017; Wang et al., 2019; Chang et al., 2020; Wang et al., 2020; Huang et al., 2021a). To confirm the causal relationship between gut microbiota and preeclampsia, gut microbiota of preeclampsia patients was transplanted into mice pretreated with antibiotics; mice receiving transplantation had higher systolic blood pressure (Chen et al., 2020). This suggests that preeclampsia patient gut microbiota may disturb blood pressure regulation. Moreover, modulation of the gut microbiota by changing daily diet has been shown to play a role in preeclampsia relief (Torjusen et al., 2014). Therefore, despite inconsistent differential intestinal bacteria reported in existing studies, exploring the relationship between gut microbiota and the pathological mechanisms of preeclampsia remains of significant importance (H., 2020).

In terms of clinical presentation, preeclampsia is a disorder of maternal blood pressure regulation that occurs during pregnancy. The gut microbiota has been shown to influence blood pressure regulation primarily through modulation of its products (see in **Table 2**) (Hsu et al., 2021). And the major bacterial metabolites, SCFAs, have been reported to be altered in patients with pre-eclampsia (Gomez-Arango et al., 2016b; Chang et al., 2020). It has been reported that the relative abundance of butyrate-producing bacteria is reduced in the gut of patients with preeclampsia, while another study confirmed that butyrate supplementation can alleviate preeclampsia lesions in rats (Altemani et al., 2021; Yong et al., 2022). SCFAs have also been proved to bind to GPR41 and GPR43 and then induce vasodilation and upregulate energy expenditure, thereby preventing fat accumulation and reducing the risk of atherosclerosis; they may also activate Olfr-78 to regulate the activity of the renin-angiotensin system, thus preventing elevated blood pressure (Hsu et al., 2021). In addition, gut dysbiosis may trigger inflammation by increasing LPS levels in the vessels and lead to oxidation of low-density lipoprotein (LDL) to ox-LDL (D, 2021). A step further, increased ox-LDL can decrease the total amount of circulating NO by impairing the activity of nitric oxide synthase and decreasing the expression of endothelin-1(ET -1), leading to hypertension (Dulak et al., 1997) (**Figure 2**). Moreover, increased TMAO levels have been confirmed in preeclampsia patients, implying that TMAO may also play a role in PE (Wang et al., 2019). Indeed, TMAO may cause disturbances in glucose and lipid metabolism and promote phagocytosis of ox-LDL by leukocytes and macrophages, leading to the formation of foam cells and increasing the risk of atherosclerosis (Hoseini-Tavassol and H-R, 2021). In addition, TMAO can also activate protein kinase C (PKC) and NF-kB pathway, which promotes macrophage adhesion and impairs the self-repair process of vascular endothelial cells (Zhang et al., 2021). It can still increase the production of reactive oxygen species (ROS) and induce the formation of NLPR3 inflammasomes in human umbilical vein endothelial cells (HUVECs), which may lead to pyroptotic injury of HUVECs (Sun et al., 2016). TMAO further exacerbates angiotension-induced hypertension II and exerts its function through the PERK/ROS/CaMKII/PLCβ3 axis (Jiang et al., 2021), which play a role in the pathophysiology of preeclampsia (Fu et al., 2015). Gut microbiota can even regulate blood pressure by altering the secretion of intestinal hormones such as GLP-1 and gastrin, thereby regulating the activity of sodium-proton exchanger subtype 3 (NHE3) and nitric oxide synthase to modulate sodium ion reabsorption in distal renal tubules and NO concentration in vascular endothelial cells, respectively (Martins et al., 2020; Vallianou et al., 2020; Younes et al., 2020). However, the effect of the intestinal hormone gastrin is much more confusing. A study comparing the serum gastrin in pregnant women with and without preeclampsia came to a negative conclusion (Morán et al., 1996), although the negative correlation is likely due to the extremely small sample size.

Gut microbiota may also play a role in blood pressure regulation by altering the nervous system (Wang et al., 2021). The interaction between gut microbiota and the enteric nervous system (ENS) has also been gradually revealed. SCFAs can directly stimulate intestinal mucosal receptors, promote the secretion of enteric neurotransmitters such as PYY, and regulate the activity of vagus and sympathetic nerves (Vallianou et al., 2020). Gut microbiota also metabolizes sulfur-containing amino acids and tryptophan to hydrogen sulfide (H_2_S) and γ-aminobutyric acid (GABA); these molecules can act as messengers to influence nervous system function (S., 2018). In addition, studies have shown that gut dysbiosis can lead to increased permeability of the blood-brain barrier (BBB), and this change promotes the influx of LPS into the central nervous system, causing neuroinflammation (Tang et al., 2020). This may also contribute to neuroinflammation and blood-brain barrier disruption reported in preeclampsia patients (Bergman et al., 2021). Interestingly, in animal studies, injection of a proinflammatory factor into the brain increased sympathetic nervous system activity and blood pressure, whereas injection of an anti-inflammatory factor such as IL -10 had the opposite effect (Shi et al., 2010). H_2_S was also reported to exert a protective effect against neuroinflammation (Ghanbari et al., 2019). In addition, the gut microbiota has been reported to play a role in immune system dysregulation in preeclampsia. Studies have demonstrated that in preeclampsia patients, the ratio of Th1 and Th2 differs from that of normal pregnant women; and the imbalance in Th1/Th2 ratio leads to a pro-inflammatory microenvironment at the maternal-fetal interface and contributes to the occurrence of preeclampsia (Vargas-Rojas et al., 2016). Modulating the gut microbiota can affect the Th1/Th2 ratio and M2 macrophage polarization (Song et al., 2020); this may improve symptoms in preeclampsia patients and may be another effective treatment for preeclampsia, although it is still far from clinical application.

Based on the comprehensive analysis presented, it indicates that the gut microbiota plays a crucial role in the onset and progression of preeclampsia. Although the gut microbiota may influence blood pressure regulation through various mechanisms, the effect of gut microbiota on the depth of placental implantation at the initial onset of preeclampsia lacks corresponding reports, which requires further studies to more fully elucidate the relationship between gut microbiota and preeclampsia.

## 5 Gut microbiota and preterm birth

Preterm birth refers to delivery before 37 weeks of gestation, with a global incidence rate ranging from 5% to 18%. It is divided into spontaneous and iatrogenic preterm birth (W, 2020). Risk factors include lower genital tract infections, smoking, malnutrition, etc (Goldenberg et al., 2008). The current therapeutic strategy for preterm birth is to prolong gestation by inhibiting uterine contractions, and, if necessary, anti-infective treatment (Hoh et al., 2019). In fact, preterm birth is a complex disease with multiple etiologies and risk factors highly correlated with systemic inflammation and immune dysregulation (Gilman-Sachs et al., 2018). Studies have confirmed that dysbiosis in the oral cavity and lower genital tract has been shown to contribute to preterm birth (Chu et al., 2018). However, it is worth noting that the incidence of preterm birth did not always decrease after targeted treatment of oral or genital tract infections (Kim et al., 2012), suggesting that other factors might contribute to preterm birth. In fact, changes in the gut microbiota during pregnancy may influence infections, inflammation, and immune dysregulation, all of which are considered primary triggers for preterm birth.

Shiozaki et al. amplified the 16S rDNA of bacteria and used terminal restriction fragment length polymorphism (T-RFLP) to analyze differences in gut microbiota between preterm birth and term mothers; results showed lower abundance of *Clostridium* subcluster XVIII, IV and XIVa, as well as *Bacteroides*, while *Lactobacillus* was significantly increased in preterm birth mother (Shiozaki et al., 2014). Another study applying high-throughput sequencing technology reported reduced α-diversity of gut microbiota in pregnant women with preterm delivery (Hiltunen et al., 2021). In general, normal pregnancy is associated with decreasing serum LPS levels as progesterone rise and pregnancy progresses, which has been proposed as a form of self-protection (Zhou et al., 2019). And maternal serum anti-LPS IgG was negatively related to pregnancy length (Lauer et al., 2018). Mechanistically, LPS activates the uterine contraction system via increased serum prostaglandins and oxytocin levels (Okawa et al., 2001). Evidence from experimental animals showed that oral administration of *Enterococcus faecium* to pregnant mice confirmed gut microbes can migrate beyond the gut and colonize the maternal-fetal interface, altering local or systemic immunity (Jiménez et al., 2005). Gut microbiota translocation may benefit from increased intestinal permeability, which is facilitated by a normal gut microbiota maintaining gut function and permeability (Allam-Ndoul et al., 2020). Air pollution may also contribute to preterm birth via disrupting the maternal gut microbiota (Gan et al., 2022). High-throughput sequencing and non-targeted metabolomics combined revealed gut microbiota influences nutritional preterm birth and may be a potential factor for predicting preterm birth (Gershuni et al., 2021). Furthermore, gut dysbiosis may also promote preterm delivery by affecting bile acid metabolism (Ramírez-Pérez et al., 2017; You et al., 2020).

In fact, vaginal microbiota have gained more prominence in preterm birth research compared to gut microbiota, and their role in the process of preterm birth is more easily understood. During normal pregnancy, immune tolerance in the pregnant woman’s body allows for an increase in vaginal microbiota diversity in the early stages, while the relative proportion of *Lactobacilli* decreases. As pregnancy progresses, the levels of pregnancy-related hormones, particularly estrogen, rise rapidly, leading to an increase in glycogen content in the vaginal epithelium. This change helps restore the relative abundance of *Lactobacilli* to pre-pregnancy levels or higher, maintaining the acidic environment of the vagina and inhibiting the proliferation of pathogenic microorganisms (Shen et al., 2022). A study has revealed differences in the vaginal microbiota of 45 preterm and 90 term-born African American women. The results indicate that, in the preterm group, the overall diversity of the vaginal microbiota increased, with a structure more akin to bacterial vaginosis. Significant decreases in *Lactobacillus crispatus* levels and significant increases in bacterial vaginosis-associated bacterium 1 (BVAB1), *Prevotella* cluster 2, and *Sneathia amnii* were observed. In contrast, the vaginal microbiota of term-born women exhibited an absolute dominance of *Lactobacillus crispatus* (Fettweis et al., 2019). Additionally, there appears to be a certain level of crosstalk between gut microbiota and vaginal microbiota during pregnancy. A randomized controlled clinical trial conducted in Malaysia included 78 patients with recurrent vaginal candidiasis, and they were randomly assigned in a 1:1 ratio to either the oral probiotic supplementation group or the placebo control group. The results showed that 47% of the participants who received oral probiotic supplements did not experience a recurrence of vaginal candidiasis. This indicates that oral probiotic supplementation can influence the structure of the perineal and vaginal microbiota, although the study did not explore the potential mechanisms involved (Ang et al., 2022). Another study conducted in the United States on pregnant women with bacterial vaginosis who received probiotic supplementation showed significant results, despite the relatively small clinical sample size (n=16). Researchers observed that pregnant women with bacterial vaginosis had markedly different vaginal microbiome structures, while their gut microbiomes were relatively similar. However, through oral probiotic supplementation induced significant positive changes in both vaginal and gut microbial community structures. Notably, among subjects receiving oral probiotics, *Prevotella copri* abundance significantly increased in the vaginal microbiome, a phenomenon also observed in the gut microbiota (Vasundhara et al., 2021). *Prevotella copri*, found in the gut, plays a role in carbohydrate, particularly fiber, metabolism, producing short-chain fatty acids (Gong et al., 2024; Yang et al., 2024). This process is beneficial for maintaining gut microbiota balance and may influence the immune system. Bacterial vaginosis and vaginal candidiasis have been widely recognized as risk factors for preterm birth (Fettweis et al., 2019). The potential effects of oral probiotics on these conditions may, in part, result from their ability to improve and regulate the gut microbiota. However, given the limited number of relevant studies reported to date, we must maintain a cautious attitude and exercise prudent judgment regarding this conclusion.

In fact, changes in the gut microbiome may influence the occurrence of preterm birth by affecting the host’s immune system and metabolic processes. At the maternal-fetal interface, immune cells including regulatory T cells (Treg cells), natural killer cells (NK cells), and macrophages undergo significant changes and play crucial roles throughout early to late pregnancy (Giles et al., 2023). Research indicates that changes in the gut microbiota can influence the host’s immune homeostasis, including effects on the proliferation and function of Treg cells (Gao et al., 2020b). These effects are partially mediated by the metabolic byproducts of the gut microbiota—SCFAs. SCFAs possess the ability to regulate inflammatory response balance and induce immune tolerance, which appears to have a positive impact on maintaining pregnancy (Hu et al., 2019). And the reduction in serum levels of SCFAs during late pregnancy may be one of the critical factors leading to immune imbalance at the maternal-fetal interface. Conversely, the host’s intestinal mucosa regulates the secretion of substances such as mucus, immunoglobulin A (IgA), and defensins by recognizing microbial-associated molecular patterns (MAMPs) and bacterial metabolites, thereby maintaining the dynamic equilibrium of the gut microbiota (Wiertsema et al., 2021). As previously mentioned, the metabolic state during pregnancy transitions from early to late stages towards an energy storage state, maintaining higher levels of serum circulating fatty acids and glucose to ensure normal pregnancy progression. These metabolic shifts appear to be associated with increased abundances of microbial communities such as *Akkermansia* and *Bifidobacterium* (Qin et al., 2021). However, studies have indicated that excessive weight gain during pregnancy may be positively correlated with iatrogenic preterm birth (Faucher et al., 2016). Furthermore, excessive weight gain can increase abdominal pressure, potentially leading to premature cervical ripening and ultimately triggering preterm birth (Khan et al., 2023).

In summary, although current research on the relationship between gut microbiota and preterm birth is still limited, existing evidence suggests that gut microbiota may influence the occurrence of preterm birth by altering vaginal microecology, immune responses, and metabolic processes.

## 6 Gut microbiota and postpartum depression

Postpartum depression (PPD), a severe form of depression that occurs after delivery, has an incidence as high as 17.22% worldwide (Wang et al., 2021). Pathophysiologically, PPD is associated with environmental factors, genetic factors, nervous system inflammation, reproductive hormone withdrawal, neuroendocrine changes, and metabolism (Payne and M, 2019). The role of gut microbiota in depression reported in recent years suggests a new direction in the study of PPD, although research in this area is limited. Zhou et al. reported lower abundance of *Faecalibacterium, Phascolarctobacterium, Butyricicoccus*, *Lachnospiraceae* and enrichment of *Enterobacteriaceae* in the gut microbiota of PPD patients (Hou et al., 2020). Furthermore, a clinical trial revealed a modest protective effect of probiotic supplementation and improved diet on postpartum depression (Hulkkonen et al., 2021). In a mouse model of obesity-induced PPD, it was found that increased intake of dietary fiber could alleviate symptoms by remodeling gut microbiota and increasing short-chain fatty acid production (Liu et al., 2020). These findings suggest that gut microbiota may impact PPD.

Recent studies suggest that the gut microbiota may have a multifaceted relationship with postpartum mental health. The gut microbiota is involved in the interaction by altering amino acid metabolism, regulating the vagus nervous system, and remodeling its metabolites, which has been termed the “microbiota-gut-brain axis” (Margolis et al., 2021). When the balance of the gut microbiota is disrupted, a shift in tryptophan metabolism can lead to an imbalance between serotonin and kynurenine, and deficiencies of serotonin and excesses of kynurenine have been implicated in postpartum depression (Duan et al., 2018). Additionally, *Lactobacillus*, *Bifidobacterium*, and *Klebsiella* have been shown to synthesize neurotransmitters such as dopamine, serotonin, Gamma-Aminobutyric Acid (GABA), and acetylcholine in the gut to mediate the microbiota’s interaction with the nervous system (S., 2018). Changes in these neurotransmitter-producing bacteria, can alter neurotransmitter levels and ratios, which may induce postpartum depression (Payne and M, 2019). Neuroinflammation is also involved in the “microbiota-gut-brain axis”. Gut dysbiosis has been shown to induce inflammatory factors such as IL-6 and TNF-α, which may contribute to postpartum depression’s pathophysiology (Beurel et al., 2020). Moreover, inflammation induced by gut dysbiosis may compromise the blood-brain barrier (BBB) and allow for an influx of LPS and peptidoglycan, which can bind to TLR4 in the brain, triggering an inflammatory cascade that promotes postpartum depression (Payne and M, 2019). Elevated LPS levels have also been implicated in upregulating central oxytocin levels and postpartum depression pathophysiology (Kong et al., 2015; Payne and M, 2019). It is worth noting that Th17 cells in the blood and IL-17A, which have long been reported to be regulated by the gut microbiota, are positively associated with postpartum depression (Liu et al., 2020; Min et al., 2022). The hypothalamic-pituitary-adrenal (HPA) axis, which refers to the feedback network between the hypothalamus, pituitary, and adrenal glands, has been reported to participate in the crosstalk between gut microbiota and postpartum depression by regulating cortisol secretion (Redpath et al., 2019). Disturbed gut microbiota has been associated with lower cortisol levels, which has been reported in depression patients, and an association with chronic postpartum depression has also been demonstrated (Seth et al., 2016). However, the specific mechanism of the gut microbiota in the HPA axis remains to be investigated (see **Figure 2**).

Although much of the evidence cited in this review focuses on depression, we can clearly conclude that the prevention of postpartum depression requires coordination between the gut microbiota, the nervous system, and the endocrine system.

## 7 Potential of probiotics administration during pregnancy

Probiotics is a general term for bacteria that exert positive effects on their hosts when alive bacteria reach a certain threshold, namely at least 1×10^9^ Colony-Forming Units (CFU) (Hill et al., 2014). Bacteria currently used as probiotics mainly include *Lactobacillus*, *Bifidobacterium*, *Enterococcus faecalis* and *Streptococcus thermophilus* (W., 2010). Numerous studies indicate that they can generate short-chain fatty acids and influence amino acid metabolism, thereby affecting energy metabolism and immune regulation in the body (Lee et al., 2018). In addition, some bacterial cell membrane components have been shown to have a regulatory effect on the immune system (Bae et al., 2022). Although the current clinical application of probiotics is relatively limited, potential beneficial effects of probiotics on the nervous system (Pluta et al., 2020), circulatory system (Liang et al., 2021), immune system (Frei et al., 2015) and even malignancies (Zheng et al., 2021) have been demonstrated. Likewise, the use of probiotics for pregnancy complaints has been studied in detail.

Preclinical studies have consistently demonstrated that probiotics have beneficial effects on pregnancy complications (see in **Table 3**). In animal models of pre-eclampsia, probiotics therapy has been shown to lower blood pressure by upregulating the level of nitric oxide (NO) and endothelin-1 (ET-1), and remodeling the gut microbiota with increased relative abundance of *Bifidobacterium* and *Lactobacillus* (Sun et al., 2020). However, clinical trial results have been inconsistent. A US trial found that *Bifidobacteria* and *Lactobacilli* supplementation improved fasting blood glucose and insulin resistance without affecting weight gain (Kijmanawat et al., 2019). Similarly, a randomized controlled trial showed probiotic (containing *Lactobacillus acidophilus*, *Lactobacillus casei*, *Bifidobacterium bifidum*, *Lactobacillus fermentum*) supplementation improved glucose and lipid metabolism in patients with gestational diabetes (Babadi et al., 2019). In contrast, a trial in Australia found oral probiotics (*Lactobacillus rhamnosus* and *Bifidobacterium animalis* subspecies *lactis*) did not prevent gestational diabetes in overweight/obese patients (Callaway et al., 2019). Differences may relate to probiotic selection, ethnicity, diet and culture, and publication bias. Furthermore, *Lactobacillus casei* has been shown to alleviate postpartum depression by regulating the microbiota-gut-brain axis (Yang et al., 2022). Recent reviews have concluded that single or combined probiotics reduce mortality in preterm and low birth weight neonates (Morgan et al., 2020). In general, despite the inherent variability among the subjects studied, the strategy of supplementing with probiotics to improve disease conditions appears to hold significant potential and promise.

**Table 3:** Clinical researches on the improvement of probiotics in gestational diseases.

Diseases	Subjects	Probiotics	Conclusion	Ref.
GDM	Homo	*Lactobacillus rhamnosus HN001*	Probiotics intake from 14 to 16 weeks’ gestation may reduce GDM prevalence, particularly among older women and those with previous GDM	(Wickens et al., 2017)
	Homo	*Lactobacillus acidophilus* LA-5, *Bifidobacterium* BB-12, *Streptococcus thermophilus* STY-31, *Lactobacillus delbrueckii bulgaricus* LBY-27	The probiotic supplement appeared to affect glucose metabolism and weight gain among pregnant women with GDM.	(Dolatkhah et al., 2015)
	Homo	*Lactobacillus acidophilus, L. casei, Bifidobacterium bifidum*	Probiotic supplements for 6 weeks in patients with GDM had beneficial effects on glycemic control, triglycerides and VLDL cholesterol concentrations	(Karamali et al., 2016)
	Homo	*Lactobacillus acidophilus* LA-5, *Bifidobacterium* BB-12, *Streptococcus Thermophilus* STY-31, *Lactobacillus delbrueckii bulgaricus* LBY-27	Probiotic supplement containing *L.acidophilus* LA- 5, *Bifidobacterium* BB-12, *S.thermophilus* STY-31 and *L.delbrueckii bulgaricus* LBY-2 appears to improve several inflammation and oxidative stress biomarkers in women with GDM	(Hajifaraji et al., 2018)
	Homo	*Lactobacillus acidophilus, Bifidobacterium lactis*	Better control of blood glucose can be achieved by consumption of probiotic in patients whose pregnancy is complicated by GDM	(Sahhaf Ebrahimi et al., 2019)
	Homo	*Lactobacillus acidophilus, Lactobacillus casei, Bifidobacterium bifidum, Lactobacillus fermentum*	Probiotic intake for 6 weeks to patients with GDM had beneficial effects on gene expression related to insulin and inflammation, glycemic control, few lipid profiles, inflammatory markers, and oxidative stress.	(Babadi et al., 2019)
	Homo	*Lactobacillus salivarius UCC118*	A probiotic capsule intervention among women with abnormal glucose tolerance had no impact on glycemic control.	(Lindsay et al., 2015)
	Homo	*Streptococcus thermophilus, Bifidobacterium breve, Bifidobacterium longum, Bifidobacterium infantis, Lactobacillus acidophilus, Lactobacillus plantarum, Lactobacillus paracasei, Lactobacillus delbrueckii subsp. Bulgaricus*	Supplementation with probiotics may help to modulate some inflammatory markers and may have benefits on glycemic control.	(Jafarnejad et al., 2016)
	Homo	*Lactobacillus rhamnosus* HN001, *Bifidobacterium animalis* ssp. *Lactis*	Probiotics conferred no benefits in lowering the risk of GDM	(Pellonperä et al., 2019)
	Homo	*Bifidobacterium bifidum, B lactis, Lactobacillus acidophilus, L paracasei, L rhamnosus, Streptococcus thermophilus*	Probiotics did not affect glycemic control of women with gestational diabetes mellitus.	(Nachum et al., 2024)
	Homo	*Lactobacillus rhamnosus*, *Bifidobacterium animalis subspecies lactis*	The probiotics used in this study did not prevent GDM in overweight and obese pregnant women.	(Callaway et al., 2019)
	Homo	*Bifidobacterium, Lactobacillus*	Probiotic supplements in the late second and early third trimester lowered fasting glucose and increased insulin sensitivity	(Kijmanawat et al., 2019)
	Homo	*Lactobacillus acidophilus, Bifidobacterium*	Probiotic yogurt consumption during pregnancy was effective in reducing the risk of gestational diabetes mellitus in Chinese women	(Chen et al., 2019)
	Homo	*Lactobacillus acidophilus, Lactobacillus casei subsp., Lactobacillus lactis, Bifidobacterium bifidum, Bifidobacterium infantis, Bifidobacterium longum*	Multi-strain probiotics are beneficial for improved metabolic and inflammatory outcomes in post-GDM women by modulating gut dysbiosis	(Hasain et al., 2022)
Preeclampsia	Homo	*Lactobacillus acidophilus* (LA-5), *Bifidobacterium lactis* (Bb12), *Lactobacillus rhamnosus GG* (LGG)	Probiotic intake in late pregnancy (but not before or in early pregnancy) was significantly associated with lower preeclampsia risk.	(Nordqvist et al., 2018)
	Homo	*L. acidophilus* LA-5, *B. lactis* Bb12, *L. rhamnosus* GG, *L. acidophilus* LA-5, *B.lactis* Bb12	Probiotics could be associated with lower risk of preeclampsia in primiparous women	(Brantsaeter et al., 2011)
PPD	Homo	*Lactobacillus rhamnosus* HN001	This probiotic may be useful for the prevention or treatment of symptoms of depression and anxiety postpartum.	(Slykerman et al., 2017)
	Homo	*Limosilactobacillus reuteri* PBS072, *Bifidobacterium breve* BB077	Probiotics supplementation is able to improve stress resilience in the postpartum period.	(Vicariotto et al., 2023)
Preterm birth	Homo	*Lac acidophilus, Lac Plantarum, Lac frementum, Lac gasseri*	The rate of preterm delivery was lower in the oral probiotic group	(Solgi et al., 2022)
	Homo	*C. butyricum, E. faecium, B. subtilis*	Probiotics may reduce the rate of recurrent spontaneous preterm delivery	(Arai et al., 2022)
	Homo	Lactobacillus acidophilus LA-5*, Bifidobacterium lactis* Bb12*, Lactobacillus rhamnosus*	Probiotic dairy products reduced risk of spontaneous preterm delivery.	(Myhre et al., 2011)
	Homo	*L.acidophilus, L.plantarum, L.fermentum, L.Gasseri*	Probiotic supplementation from the 16th week until 37th of pregnancy can reduce preterm birth.	(Vanda et al., 2024)

## 8 Conclusions and future directions

The study of gut microbiota has opened new avenues for understanding the etiology and pathology of various diseases, including during pregnancy and the postpartum period. Although current research on the relationship between gut microbiota and disease during pregnancy shows promise and is popular, many findings may not have an immediate visible effect and further research is warranted.

First, gut microbiota composition exhibits significant heterogeneity, influenced by differences in genetic background and dietary habits. Second, various clinical studies differ in sample collection, sequencing technology, and sequencing depth, which may be primary drivers of inconsistent findings across studies on the same disease. In addition, current dietary and probiotic interventions are not effective for all patients, and trial results are inconsistent. This poses a challenge to the study of gut microbiota, highlighting the need for further experimental reviews with greater stability and reproducibility. One potential approach is to develop tailored probiotic strains for specific populations based on their dietary and cultural characteristics, thereby reducing variability in experimental results when probiotics are applied to different groups. Furthermore, to clarify the regulatory network of gut microbiota in multiple physiological systems, upcoming research should prioritize mechanistic exploration. This is crucial for effectively curbing disease progression and simultaneously reducing medical costs.

In summary, published literature suggests that gut microbiota plays a vital role in the initiation, development, prevention, and treatment of diseases during pregnancy. The application of probiotics in gestational diseases is promising. However, the use of gut flora modulation as a treatment strategy still requires comprehensive and multifaceted assessments of disease-specific bacterial strains to optimize interventions and maximize therapeutic outcomes.
